# The future of cancer immunotherapy: microenvironment-targeting combinations

**DOI:** 10.1038/s41422-020-0337-2

**Published:** 2020-05-28

**Authors:** Yonina R. Murciano-Goroff, Allison Betof Warner, Jedd D. Wolchok

**Affiliations:** 1 Department of Medicine, Memorial Sloan Kettering Cancer Center, New York, NY 10065 USA; 2 Human Oncology and Pathogenesis Program, Memorial Sloan Kettering Cancer Center, New York, NY 10065 USA; 3 Weill Cornell Medicine, New York, NY 10065 USA; 4 Parker Institute for Cancer Immunotherapy, San Francisco, CA USA

**Keywords:** Cancer microenvironment, Cancer immunotherapy

## Abstract

Immunotherapy holds the potential to induce durable responses, but only a minority of patients currently respond. The etiologies of primary and secondary resistance to immunotherapy are multifaceted, deriving not only from tumor intrinsic factors, but also from the complex interplay between cancer and its microenvironment. In addressing frontiers in clinical immunotherapy, we describe two categories of approaches to the design of novel drugs and combination therapies: the first involves direct modification of the tumor, while the second indirectly enhances immunogenicity through alteration of the microenvironment. By systematically addressing the factors that mediate resistance, we are able to identify mechanistically-driven novel approaches to improve immunotherapy outcomes.

## Introduction

In addition to surgery, chemotherapy, targeted pathway inhibition and radiation therapy, immunotherapy has emerged as a standard pillar of cancer treatment. Immune checkpoint inhibitors (ICIs) such as those targeting cytotoxic T lymphocyte-associated protein 4 (CTLA-4) and programmed cell death protein 1/programmed cell death ligand 1 (PD-1/PD-L1) have been integrated into standard of care regimens for patients with advanced melanoma, Merkel cell carcinoma, non-small cell lung cancer, cutaneous squamous cell carcinoma, urothelial cancer, renal cancer, refractory Hodgkin lymphoma, hepatocellular carcinoma, gastric cancer, triple-negative breast cancer, and microsatellite instability (MSI)-high tumors. Beyond checkpoint inhibitors, cellular therapy in the form of chimeric antigen receptor (CAR) T cells directed at CD19 are now approved in patients with refractory B cell acute lymphoblastic leukemia and large B cell lymphoma. Novel indications and integration of immunotherapy into earlier stages of disease are being actively investigated (Table 1).

**Table 1:** Summary of FDA-approved immunotherapies.

Mechanism	FDA-approved therapies	Disease indication (year of approval)
Anti-CTLA4	Ipilimumab	•Melanoma (2011) •Renal cell carcinoma (2018) •MSI-H or dMMR colorectal cancer (2018) •Hepatocellular carcinoma (2020)
Anti-PD1	Nivolumab	•Melanoma (2014) •Non-small cell lung cancer (2015) •Renal cell carcinoma (2015) •Hodgkin lymphoma (2016) •Squamous cell of the head and neck (2016) •Urothelial carcinoma (2017) •MSI-H or dMMR colorectal cancer (2017) •Hepatocellular carcinoma (2017) •Small cell lung cancer (2018)
	Cemiplimab	•Cutaneous squamous cell carcinoma (2018)
	Pembrolizumab	•Melanoma (2014) •Non-small cell lung cancer (2015) •Head and neck squamous cell carcinoma (2015) •Hodgkin lymphoma (2017) •Urothelial carcinoma (2017) •MSI-H cancer (2017) •Gastric cancer (2017) •Cervical cancer (2018) •Primary mediastinal large B-cell lymphoma (2018) •Merkel cell carcinoma (2018) •Renal cell carcinoma (2019) •Esophageal cancer (2019) •Hepatocellular carcinoma (2019) •Endometrial carcinoma (2019)
Anti-PD-L1	Atezolizumab	•Urothelial cancer (2016) •Non-small cell lung cancer (2016) •Triple-negative breast cancer (2018) •Small cell lung cancer (2019)
	Avelumab	•Merkel cell carcinoma (2017) •Urothelial cell carcinoma (2017) •Renal cell carcinoma (2019)
	Durvalumab	•Urothelial cell carcinoma (2017) •Non-small cell carcinoma (2018) •Small cell lung cancer (2020)
CAR-T cell therapy	Axicabtagene ciloleucel	•Large B-cell lymphoma (2017)
	Tisagenlecleucel	•B-cell precursor acute lymphoblastic leukemia (2017) •Large B-cell lymphoma (2018)
Cytokine modulation	Interferon	Interferon Alfa-2B: •Hairy cell leukemia (1986) •AIDS-related Kaposi’s sarcoma (1988) •Melanoma (1995) •Follicular lymphoma (1997)
	Interleukin	Interleukin-2: •Renal cell carcinoma (1992) •Melanoma (1998)
Dendritic cell vaccine	Sipuleucel-T	•Prostate cancer (2010)
Oncolytic viruses	Talimogene laherparepvec	•Melanoma (2015)

Clinical enthusiasm for immunotherapy is high, largely due to the potential for durable responses, with over 2000 trials ongoing investigating anti-PD-1/anti-PD-L1 targeted drugs alone.^1^ However, it is only a minority of patients treated with immune checkpoint inhibitors (ICIs) that respond to these agents.^2,3^ A portion of those patients who do respond will go on to later have progressive, refractory disease.^4^ Primary and acquired resistance necessitates novel agents and combinations.

**Fig. 1:**
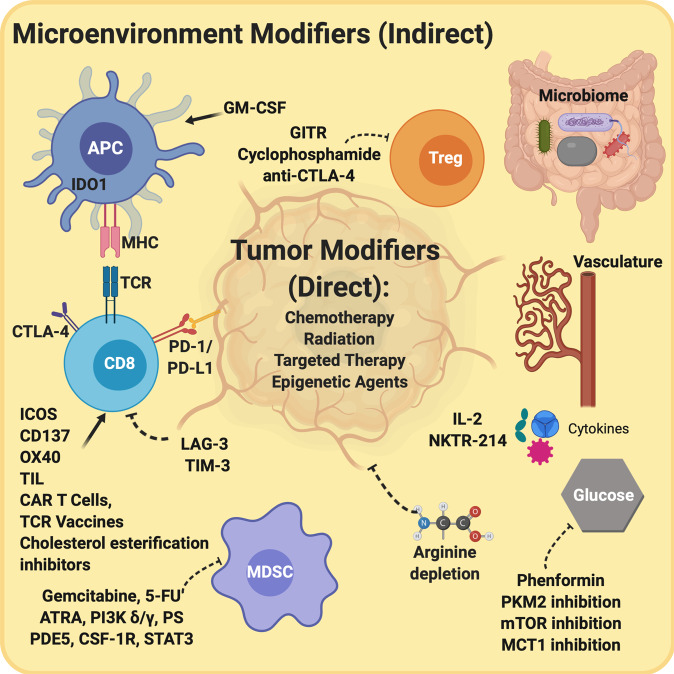
Schematic diagram of the interaction between indirect modifiers of the tumor microenvironment and direct tumor modifiers. Direct tumor modifiers act on tumor cells to promote cellular death. These strategies include chemotherapy, radiation therapy, targeted therapies, and epigenetic agents. Indirect modifiers operate predominantly to shift the microenvironment to favor anti-tumor immunity. This can be achieved by enhancing the efficacy or quantity of effector T cells and APCs and/or inhibiting tolerogenic cells such as Tregs and MDSCs. Indirect modulators may also alter the microenvironment through modification of the gut microbiome, the local vasculature, the cytokine milieu, or by altering cellular metabolism, including of amino acids, glucose, and lipids. As depicted, these mechanisms do not operate in isolation, as modification of the microenvironment may enhance direct tumor cell killing and vice versa.

Resistance to immunotherapy is multifaceted. Much attention has been paid to tumor intrinsic factors such as PD-L1 expression,^5^ mutational burden,^6^ and deficiencies in antigen presentation,^7^ but the problem of immunotherapy resistance is more complex because tumors exist in a dynamic microenvironment. The tumor microenvironment is a milieu of malignant cells, immune components, blood vessels, extracellular matrix, and signaling molecules that work individually and in combination to influence sensitivity to immunotherapy. Here, we review a variety of strategies to modulate the microenvironment with the goal of enhancing response to immunotherapy. The approaches fall into two broad categories: direct and indirect modulation of immunogenicity. Direct approaches primarily modify the tumor itself, whereas indirect approaches operate predominantly on the microenvironment (Fig. 1). These two categories of approaches are inextricably linked, with direct modification of the tumor often leading to changes in the microenvironment and vice versa. We suggest these categorizations as a means to enhance understanding of the primary goal of a particular strategy, and we posit that rational combinations of microenvironment-targeting therapies with ICI or cellular therapy will comprise the next generation of immune-based approaches to cancer treatment.

## Indirect modulation of tumor immunogenicity

### Markers of an immunogenic microenvironment

To optimize the tumor microenvironment, we must first understand what defines favorable conditions. Immune cells are necessary for anti-tumor response, but their presence is not sufficient; other mediators play key determining roles. Effector CD8^+^ T cells (Teff) compete with anti-inflammatory cytokines and cells promoting immune tolerance, including myeloid-derived suppressor cells (MDSC) and regulatory T cells (Tregs). The Teff/Treg ratio is a prognostic and predictive marker in many tumor types.^8,9^

Solid tumors have been classified as “inflamed” (highly infiltrated with immune cells and proinflammatory cytokines), “immune-deserts” (minimal effector immune cell infiltrate) or the intermediate “immune excluded” (immune cells present in the stroma but not the tumor parenchyma).^10^ Inflamed tumors, unsurprisingly, are associated with better clinical outcomes. However, CD8^+^ cell infiltration into the tumor is at best an imperfect marker of immunogenicity, and not all patients with the inflamed phenotype respond to immunotherapy. In melanoma patients, baseline CD8^+^ levels within the tumor are associated with response to PD-1 therapy, whereas with the anti-CTLA-4 agent ipilimumab, response is better correlated with post-treatment increases in tumor-infiltrating lymphocytes (TILs) rather than baseline levels.^11–13^ This is to say, an inflamed tumor phenotype can promote response, but treatment-induced modulation of a less immunogenic tumor may yield similar results, highlighting opportunities for therapeutic intervention.

While defining the immunogenicity of a tumor and its microenvironment is challenging, clinical studies have validated several biomarkers. Tumor intrinsic factors including PD-L1 expression,^11^ tumor mutation burden,^6^ and mismatch repair deficiency^14^ are clinically useful, yet imperfect biomarkers because they center around tumor cells. We now recognize the pivotal role of the microenvironment, and emerging predictors of responsiveness to anti-PD-1 immunotherapy include associations with T cell receptor (TCR) diversity and/or clonality,^13,15^ host HLA genotype,^16^ a favorable gut microbiome,^17,18^ and even body mass index,^19–22^ possibly mediated by leptin, among others.

### Stimulating a more robust T cell response to the microenvironment

Strategies designed to increase native T cell activation in response to local triggers are at the forefront of cancer immunotherapy. T cell responses are heavily regulated to ensure balance between enabling immune reactivity to foreign antigens and safeguarding against unchecked inflammation. This balance is carefully maintained by co-stimulatory and co-inhibitory molecules.^23^ Expression of co-inhibitory molecules by tumors alters the balance of the tumor microenvironment, tipping the scales toward immune suppression by increasing Treg infiltration and decreasing Teff activity.^24^ Therapeutic interventions relying on blockade of co-inhibitory molecules and/or augmentation of co-stimulatory molecules to establish a pro-immunogenic tumor microenvironment form the foundation of current immunotherapy strategies (Fig. 1).

#### Blocking inhibitory checkpoints

PD-1 and CTLA-4 are the best known of the class of immune checkpoints, which abrogate T cell reactivity to cancers and are targeted by tumors to disable anti-tumor immunity. Building on the success of CTLA-4 and PD-1/PDL-1 inhibitors, blockade of alternative immune checkpoints is an area of extensive preclinical and clinical investigation.

ICIs directed at lymphocyte-activation gene 3 (LAG-3), a cell surface molecule expressed on Teff and Tregs, are among the most heavily studied to date. Binding of LAG-3 to its primary ligand MHC class II results in suppression of Teff activity in a manner similar to PD-1 and upregulates Treg activity, creating a tolerizing microenvironment for tumor growth.^25^ Inhibition of LAG-3 has shown synergy with PD-1 inhibition in mouse models and enabled more robust T cell responses to stimulation with dendritic cell toll-like receptor (TLR) vaccination, suggesting that co-signaling blockade could restore a favorable immune microenvironment that can respond to antigenic stimulation.^26,27^ At least 60 clinical trials are currently ongoing targeting LAG-3 both alone and in combination with other immune checkpoints, with results eagerly awaited.

Another heavily studied inhibitory checkpoint mediating T cell exhaustion is the T-cell immunoglobulin and mucin-domain-containing molecule 3 (TIM-3), which is expressed on numerous types of immune cells.^28^ TIM-3 also has numerous known ligands; binding to galectin 9 leads to T cell death, while CEACAM1 binding appears to promote tolerance, although paradoxically CEACAM1-deficient animal models show increased tumor growth. Other TIM-3 ligands, including phosphatidylserine (PtdSer) and HMGB1, are thought to be especially important for antigen sensing and presentation by dendritic cells.^28^ Upregulation of TIM-3 on TILs has been correlated with poor outcomes in multiple different types of cancer,^29–32^ while anti-TIM-3 appears to enable IFN-γ-producing CD8^+^ T cell activity,^33^ though with the potential to exacerbate auto-immune-mediated reactions, including pneumonitis.^34^ Preclinical models of lung cancer suggest that TIM-3 upregulation may be a mechanism of acquired resistance to PD-1 blockade.^35^ Combinations of TIM-3 inhibition with anti-CTLA-4 or anti-PD-1 have demonstrated preclinical efficacy, and numerous clinical trials are ongoing.^28,33,35^

#### Activating stimulatory pathways

Inhibitory checkpoints are part of a much larger picture. Absence of co-stimulation can also lead to ineffective immune responses to tumor antigens. Theralizumab (TGN1412), a monoclonal agonist of CD28, served as a cautionary first attempt to manipulate co-stimulatory molecules to enhance tumor response, leading to severe cytokine release syndrome with multi-organ dysfunction in several participants.^36^ The severity of toxicity and poor understanding of its underlying mechanism dampened enthusiasm for additional co-stimulatory strategies, but recent breakthroughs have reinvigorated this field.

Several attractive co-stimulatory targets have emerged, including ICOS (inducible T-cell costimulator), CD40, TLRs, and OX40.^37^ ICOS is a member of the CD28-superfamily that is inducibly expressed on activated T cells and modulates a variety of T cell functions, including Teff activation, interactions with B cells, and Treg infiltration.^37^ Preclinical work suggests that an ICOS agonistic aptamer increases the efficacy of anti-CTLA-4 therapy against melanoma in vivo.^38^ Agonist monoclonal antibodies to ICOS are currently in early phase clinical trials together with anti-PD-1 therapy for solid tumors.^39^ OX40, is a member of the TNF receptor superfamily that promotes activation, survival, proliferation and effector function of T cells.^40^ Although OX40 is implicated in Treg development, agonism of OX40 inhibits Treg function. As a single agent, OX40 weakly enhances immunogenicity, but combinatorial strategies to enhance the T cell repertoire and agonize the immune microenvironment are thought to have more promise.^40–42^ Thus far, clinical trials of OX40 combinations have shown limited efficacy. In addition to trials using co-stimulatory agonists as primary therapies, one promising use of co-stimulatory agonists is to prolong longevity and functionality of adoptive T cell therapies.^43^

As key effectors of immune activation, enhancement of cytokines is a promising approach. In fact, some of the earliest immunotherapy strategies involved exogenous administration of interferon and Interleukin-2 (IL-2). Both therapies exhibited only modest efficacy and caused significant toxicity, limiting their clinical utility. However, improvements in engineering have triggered renewed interest in cytokine administration to enhance native T cell response. For example, NKTR-214, is a pegylated version of IL-2 designed to induce less toxicity than the IL-2 cytokine alone, and this formulation was safe in a phase I trial.^44^ On-trial biopsies demonstrated an increase in immune infiltrate with a shift toward an effector phenotype. Several trials of NKTR-214 with PD-1 pathway blockade (e.g., NCT03138889) or with CTLA-4/PD-1 combination blockade are currently enrolling (NCT02983045).

Another avenue to enhance native T cell responses derives from an appreciation of spatial relationships within the tumor microenvironment. Bispecific antibodies simultaneously engage T cells, while also binding other immune effectors.^45^ The earliest success from this strategy has been seen in patients with B-cell leukemia using the bispecific blinatumomab, which binds CD19 on the tumor cell and CD3 on the T cell.^46^ Bispecific antibodies are being designed to bind co-stimulatory and co-inhibitory molecules, including PD-(L)1, 4-1BB, and LAG-3 among others, which could facilitate direct enhancement of the microenvironment.

Finally, agonists designed to enhance innate immunity are being developed in parallel with adaptive immune stimulants. TLRs direct the activities of innate immune cells and alter cellular metabolism.^37^ Several therapies using TLR agonists as well as antagonists are currently in early phase trials. For example, the TLR9 agonist CMP-001 is being tested as monotherapy and in combination with checkpoint inhibitors for a variety of solid tumors (e.g., NCT03983668, NCT02680184, NCT03618641, etc.). Similarly, the TLR9 agonist SD-101 is being studied in combination with anti-PD-1 therapy (NCT02521870) as well as combined with targeted therapy and radiation (NCT02927964). TLR7 is also being targeted in combination with chemo-RT as well as with ICI (NCT03276832, NCT01421017).

#### Vaccines as modulators of the immune microenvironment

The goal of cancer vaccines is to increase immune responses via direct antigen injection or lysis of cancer cells to expose intratumoral antigens.^47^ Vaccines can come in the form of cells, peptides/proteins, viruses or DNA/RNA. Cell-based vaccines utilize inactivated whole tumor cells from an individual patient as antigens. Sipuleucel-T, a dendritic cell vaccine for castrate-resistant prostate cancer, was the first FDA-approved cell-based vaccine, but others including OncoVAX and GVAX are being actively investigated.^48,49^ Expense and difficulty involved in producing vaccine from individual patients limit the usefulness of this approach. Protein/peptide vaccines are synthesized as 20–30 amino acid sequences containing the specific epitope of an antigen combined with an adjuvant. Peptide-based vaccines are more easily made, more stable than cell-based vaccines, and are safe, but their performance in clinical trials has been disappointing to date.^50^ In genetic vaccines, DNA or RNA can be packaged, taken up by antigen-presenting cells (APCs), and translated into tumor-specific antigens. Effective delivery of the genetic material can be problematic, decreasing the subsequent translation of the protein and antigen presentation.^51^ Electroporation or use of viral vectors could enhance delivery, but clinical applicability of these approaches have been limited.^48^

Recent efforts have been directed at synthesizing personalized vaccines by focusing on neoantigens (also known as tumor-specific antigens) created by nonsynonymous mutations or errors in transcription in cancer cells.^52^ Next-generation sequencing can be used to identify neoantigens by comparing tumor cells to peripheral blood mononuclear cells, because neoantigens are specific to tumor cells. To create a personalized cancer vaccine, one can combine DNA, RNA, or peptides encoding the target antigen with an adjuvant to enhance immune response. Once taken up, the neoantigen is carried to the lymph node by an APC and presented to T lymphocytes, causing neoantigen-specific T cells to expand and migrate to the tumor.

Proof of concept studies have demonstrated the feasibility and potential efficacy of this approach. In a phase I study using a dendritic cell containing neoantigens with high binding affinity for HLA-A2, vaccination resulted in expansion of CD8^+^ T cells specific to about half of the immunogenic peptides.^53^ Anti-tumor activity was not assessed. More recently, computational modeling was used to identify neoantigens likely to bind HLA-A or HLA-B proteins in patients with high-risk melanoma.^54^ Six patients were vaccinated with up to 20 personalized neoantigens in the adjuvant setting, with four patients free of recurrence at a median of 25 months follow-up.

An RNA-based neoantigen vaccine strategy was also developed by identifying neoantigens and screening for binding affinity to MHC. Initial studies demonstrate robust T cell responses against the neoantigens.^55^ Five patients with metastatic disease were treated, two of whom had an objective response to vaccine-alone and one of whom had a complete response to a combination of vaccine plus anti-PD-1 therapy. Though these are small studies, the compelling results suggest that personalized cancer vaccines are likely to be a potent constituent of future immunotherapy regimens.

#### Oncolytic viruses

An alternative strategy to enhance tumor antigen recognition and strengthen T cell response is to introduce oncolytic viruses directly into the tumor microenvironment. Oncolytic viruses preferentially infect or replicate in tumor cells.^56^ Downstream anti-tumor effects include (1) inducing tumor cell lysis via intracellular proliferation, (2) releasing cytokines and viral pathogen-associated molecular patterns (PAMPs) that enhance CD8^+^ T cell activation, and (3) NK cell-mediated innate immune responses.^56^ Effects are seen not only locally, but increased CD8^+^ and tumor-specific CD4^+^ cells have been reported at distant sites.^57^

The first FDA-approved oncolytic virus, talimogene laherparepvec (T-VEC), has multiple immune-enhancing microenvironmental effects.^58^ T-VEC is a modified herpes simplex virus injected intra-lesionally for patients with unresectable melanoma. It causes direct lysis of tumor cells and releases granulocyte-macrophage colony-stimulating factor (GM-CSF), which acts as a cytokine that promotes APC recruitment, maturation, and function. T-VEC has demonstrated robust T cell responses at the injected site and distant tumor sites.^59,60^

Combination trials with oncolytic viruses represent an active frontier of immuno-oncology. T-VEC combinations with checkpoint inhibitors show early promising results in melanoma.^61,62^ Of note, in a trial of T-VEC plus pembrolizumab, responses did not correlate with baseline CD8^+^ T cell infiltration or interferon-gamma expression, but objective responses were associated with post-treatment infiltration and expression, indicating that T-VEC may modify the microenvironment to enhance PD-1 response.^63^

A similar strategy has been employed to create adenovirus-based ONCOS-102. Adenoviruses activate the innate immune system, including dendritic cells. ONCOS-102 consists of adenovirus engineered to express GM-CSF, which showed preliminary anti-tumor efficacy as a monotherapy.^64^ ONCOS-102 has been combined with cyclophosphamide in order to upregulate pro-inflammatory immune components and deplete the microenvironment of immunosuppressive cells.^65^ ONCOS-102 is currently being studied with cyclophosphamide plus other anti-cancer therapies in melanoma (NCT03003676), prostate cancer (NCT03514836), and advanced peritoneal malignancies (NCT02963831).

Additional oncolytic viruses are being explored, including vaccinia virus, measles virus, coxsackie virus, and poliovirus among others.^66^ Targeting other components of the microenvironment may also enhance the efficacy of oncolytic viruses. For example, oncolytic viruses have been shown to further disrupt tumor vasculature, which is known to be abnormal and poorly functional in cancers at baseline.^67^ Preclinical data suggest that pharmacologically improving tumor blood vessel structure can improve responses to oncolytic viruses, including immune cell trafficking into the tumor, tumor shrinkage, and decreasing metastasis.^68^

#### Epigenetic modification

Epigenetic changes within cancer cells may abrogate immune recognition, at least in part by downregulating expression of potent tumor antigens. A classic example is the cancer-testis/cancer germline antigen, NY-ESO-1, which is only expressed in germ cells, placenta, and some tumor histologies.^69^ Its potent immunogenicity makes NY-ESO-1 an appealing immunotherapy target, but its expression is heterogeneous within and between tumors. NY-ESO-1 expression is negatively regulated by DNA methylation and positively regulated by histone acetylation.^69–71^ Demethylating agents increase NY-ESO-1 expression in tumor cells and induce CD8^+^ cell immune responses in preclinical models.^72^ Other antigens, such as MAGE-A1 and endogenenous retroviruses, may also be susceptible to modulation by DNA methyltransferase inhibitors.^73^

In pre-clinical ovarian cancer models, epigenetic regulation of chemokines alters T-cell trafficking, mediated in particular by Th1-type chemokines CXCL9 and CXCL10.^74^ Combining epigenetic modulators such as DNA methyltransferases and/or histone deacetylase (HDAC) inhibitors with ICI or other immunotherapy may be a promising approach.^75^ Combination strategies with hypomethylating agents, such as 5-aza-2′-deoxycytidine, are also being studied for their role in upregulating PD-1 through induction of demethylation, along with PD-L1 and CTLA-4 inhibition.^73^

Epigenetic modifiers may serve a dual function in the tumor microenvironment by enhancing the effector function of T cells in addition to antigen expression. Reinvigoration of exhausted T cells by ICI is associated with significant chromatin remodeling, suggesting that epigenetic manipulation may benefit some patients with exhausted T cell function.^76^ HDAC inhibitors inhibit activation-induced cell death (AICD) of T cells, and preclinical models indicate that HDAC inhibitors with ICI may prevent T cell death and enhance anti-tumor responses.^77^ Clinical trials utilizing this approach are enrolling.

Epigenetic regulators may also mediate the activities of other inflammatory cells. For example, IFNγ appears to both promote a macrophage inflammatory phenotype and suppress anti-inflammatory signals through epigenetic macrophage regulation.^78^

### Hampering immune tolerance

Immune activation is counterbalanced by elements of the microenvironment that promote immune tolerance and prevent uncontrolled inflammation. Tumors coopt these mechanisms to suppress anti-tumor immunity. In addition to inhibitory checkpoints such as PD-1 and CTLA-4, key cellular mediators of tumor immune tolerance are MDSCs, Tregs, tumor-associated macrophages, and defective APCs (Fig. 1).^79^

MDSCs are cells of the mononuclear myeloid lineage that arise in conditions of chronic inflammation such as cancer to protect against tissue damage. MDSCs produce nitric oxide, cytokines, and reactive oxygen species to inhibit T cells through antigen-specific and nonspecific mechanisms. In addition to directly suppressing T cells, MDSCs promote angiogenesis, metastasis and resistance to chemotherapy, targeted therapy, and immunotherapy.^80^ Higher MDSC infiltration correlates with poor overall survival (OS) and progression-free survival (PFS) in patients with solid tumors.^79^

In healthy individuals, Tregs are critical mediators of self-tolerance, which abrogate the activity of APCs and effectors cells. Like MDSCs, high concentration of tumor-infiltrating Tregs is associated with poor prognosis in many solid tumors.^81^ Tregs suppress T cell responses in large part by binding IL-2 with high affinity, thereby limiting IL-2 availability for effector cells.^82^ They also express CTLA-4 and produce immunosuppressive cytokines, including IL-10.^81^ Indeed, murine models indicate that CTLA-4 blockade selectively depletes Tregs in the tumor microenvironment via antibody-dependent cellular toxicity.^83,84^ This may be an important mechanism by which CTLA-4 blockade exerts anti-tumor effects. CTLA-4-mediated Treg depletion increases the Teff/Treg ratio within the tumor microenvironment and is associated with enhanced effector phenotypes of CD8 and CD4 cells in the tumor microenvironment.^85^

Aside from CTLA-4, minimizing the presence or activity of suppressive cells in the tumor microenvironment is an active area of research (Fig. 1). Several FDA-approved agents have been associated with reduced MDSCs in cancer patients, including the PDE5-inhibtor tadalafil and all-trans-retinoic acid (ATRA).^80^ In animals, modest doses of the cytotoxic agents gemcitabine and 5-fluorouracil or agonism of TNF-related apoptosis-induced ligand (TRAIL) receptors can deplete MDSCs and improve anti-tumor immunity.^86–88^ PI3K-γ, CSF-1R, STAT3, and phospholipid phosphatidyl serine (PS) are other targets of exploration to selectively inhibit MDSCs.^89–93^ Liver X receptor (LXR) induction has also been associated with MDSC depletion in vivo and in vitro,^94^ and the LXRβ agonist RGX-104 is currently being evaluated in a phase I clinical trial (NCT02922764).

With respect to Tregs, glucocorticoid-induced tumor necrosis factor receptor-related protein (GITR) is being targeted to downregulate Treg function,^95^ and these antibodies are currently under investigation in combination with PD-1 inhibition.^96^

### Changing the T cell milieu: adoptive cellular therapies

While many strategies focus on modifying the native tumor microenvironment, an alternative strategy involves the direct infusion of engineered immune cells or cellular receptors into the patient via adoptive cellular therapy with the goal of increasing the number of effector cells that recognize tumor-expressed antigens. Infusion of exogenous cells or cellular components may have several advantages over efforts to augment the responses of native immune cells alone. Lymphocytes cultured ex vivo are not continually exposed to tolerogenic microenvironmental signals and may offer more potent responses to tumor antigen upon reinfusion. Moreover, adoptive cellular therapy enables the selection and expansion of cells or cell components directed against specific antigens, thereby concentrating the effectors that are most relevant to tumors.^97^ Three major types of adoptive T cell therapy are currently being studied, including TIL treatment, CAR T cell therapy, and TCR-engineered cell therapy.

#### TIL infusions

Increased levels of TILs correlate with a favorable microenvironment and improved prognosis in several studies across multiple tumor types.^98^ This approach consists of harvesting and culturing lymphocytes from excised tumor tissue, followed by testing for responsiveness to specific neoepitopes. Lymphocytes predicted to recognize tumor antigens with high affinity are expanded ex vivo and reinfused into the patient. Initial TIL studies involved solely infusion of tumor antigen-specific lymphocytes and demonstrated only modest efficacy. More recent trials have coupled TIL infusions with efforts to manipulate the host microenvironment through preparative and post-infusion regimens so as to promote TIL activation and proliferation after transfer.^97,99^ Pre-infusion lymphodepletion with chemotherapy (fludarabine or cyclophosphamide) or total body irradiation has been effective.^100^ IL-2 is given post-TIL infusion to sustain in vivo expansion but can be complicated by flu-like symptoms and capillary leak syndrome. Recent trials have aimed to better define the minimal dose of IL-2 necessary, but toxicities related to the preparative regimen and immune-related adverse events remain challenging.^101,102^ Patients with poor performance status may have difficulty tolerating TIL therapy. Moreover, successful TIL harvest can be problematic, particularly in the setting of systemic therapy. In one study, TIL harvest was unsuccessful in approximately half of the patients who attempted harvest within 30 days of systemic therapy.^103^

Early trials in melanoma suggest some promise. Three trials in 93 patients with metastatic melanoma treated with autologous TIL therapy plus IL-2 infusion showed overall response rates between 49% and 72%, with durable responses in 19 patients.^97,102^ A phase I trial of TIL therapy in heavily pre-treated patients with melanoma is ongoing, and the regimen has shown safety including in patients with prior anti-PD-1 exposure.^99^ Computational methods to better define neoepitopes may enable TIL therapy in less inflamed tumors.^104^

#### CAR T cell therapy

Whereas TIL therapy involves selection and proliferation of native tumor lymphocytes, both CAR T cell and TCR therapies involve the exogenous selection of T cell components with the potential to promote an immunogenic microenvironment. These components are engineered and expanded ex vivo and infused into the patient. CARs are created following collection of patient T cells through apheresis and engineered products are reinfused after a preparative regimen. CAR T cells couple an extracellular antibody-derived receptor designed to specifically interact with a tumor antigen with a CD3-based intracellular activating domain.^105^ Second generation CAR T cells rely on molecular components of co-stimulatory molecules, such as CD28, ICOS, OX40 and 4-1BB, to enable sustained responses in the face of repeated antigenic stimulation.^106^

CAR T cells have demonstrated success in hematologic malignancies, and CAR T cells targeting CD19 have been FDA-approved for use including for treatment of B-ALL, as well as in certain B-cell lymphomas. Axicabtagene ciloeucel is coupled to a CD28 co-stimulatory molecule, and Tisagenlecleucel is coupled to a 4-1BB co-stimulatory domain.^107–111^ Other targets are being studied, both to expand the range of tumors that can be targeted with CAR T cell therapy as well as to address resistance to CARs mediated by alterations in antigen expression.^112,113^

The creation of a more immunogenic microenvironment using CAR T cells has come at the cost of significant toxicity. Cytokine release syndrome is seen in roughly 55% of patients and neurotoxicity in 30%–40%.^114^ Treatment-related mortality may be as high as 15% among patients treated with CAR T cells, though improved recognition and management of toxicities is expected to improve the risk.^115^ Waiting for CAR T production also poses a risk for patients.^116^ Furthermore, CAR T cell manufacturing fails in up to 9% of patients.^117^ Preliminary research aimed at producing “off-the-shelf” universal CAR T cell products using gene editing designed to address manufacturing delays is ongoing.

Despite success in hematologic malignancies, solid tumors have posed a greater challenge for CAR T cells. Target identification, as well as microenvironmental suppressors such as hypoxia, PD-1 expression, and Tregs, all complicate CAR T cell therapy for solid tumors.^112^ Combinatorial strategies to generate a more favorable microenvironment are being studied with CAR T cells in this setting.

#### TCR therapy

TCRs can also be engineered, expanded ex vivo, and reinfused so as to alter the T cell component of the microenvironment. The structures of CAR T cells and TCR therapies differ, as CAR T cells harbor intrinsic signaling capacity.^106^ TCRs can only respond to MHC-presented peptide antigens, unlike CAR T cells that can recognize antigens without MHC. This distinction is important, since tumor cells often promote immunogenic tolerance through downregulation of MHC.^7^ However, an advantage of TCRs exists in their ability to respond to less dense antigens within the tumor environment and recognize antigens expressed both intracellularly and extracellularly, whereas CAR T cells can only recognize membrane-expressed antigens.^106^

The hypothetical versatility of TCR therapy is attractive, because TCRs can be engineered to respond to a variety of stimuli. However, on- and off-target toxicities have made target identification challenging. For example, targeting of MART-1 led to ocular, skin, and ototoxicities due to the presence of these epitopes in normal tissue structures, and trials with MAGE-A3 led to significant neurotoxicity, cardiac toxicity, and patient deaths.^118–120^ Though the potential toxicity is concerning, early efficacy data are promising. A phase I/II of an NY-ESO-1-specific TCR yielded an 80% response rate.^121^ Viral signatures and neoantigens identified by mutational analysis represent promising possible targets that are potentially highly specific and may minimize off-target reactivity.

### Altering the Gut microbiome

Targeting the cellular components of the microenvironment is not the only way to modify immunogenicity. Another emerging factor is the gut microbiome (Fig. 1). Patients harbor disparate compositions of gut flora by virtue of a number of primarily environmental factors, including dietary habits and antibiotic exposure. While gut bacteria like *H. Pylori* are known to mediate carcinogenesis, recent data indicate that bacteria may also alter responses to cancer therapy including ICI.^122^ Theorized mechanisms include cross-reactivity between tumor and microbial antigens as well as the role of the microbiome in enhancing dendritic cytokine release in the gut, altering the activation of circulating lymphocytes.^123^ In mice treated with anti-CTLA-4 therapy, anti-tumor responses required the presence of specific bacterial species.^124^ Antibiotic-treated mice, in particular, did not respond to anti-CTLA-4 blockade, while those who had received a bacterial gavage appeared to have restored responses. Similarly, oral Bifidobacterium administration augments the efficacy of anti-PD-L1 therapy in mouse melanoma models.^125^

Analyses of patient stool has shown that specific bacterial species are increased in responders to immunotherapy, including *Bifidobacteria, Enterococci, Akkermansia*, and *Ruminococci*.^17^ When stool bacteria from responders to immunotherapy is given to tumor-bearing mice, improved ICI responses are seen.^17^ Notably, studies aimed at identifying the specific bacterial species contributing to response and/or toxicity have generated discordant results, possibly due to diverse means of analyzing the composition of patients’ gut microbiota.^126,127^ It is more widely accepted that the overall diversity of gut bacteria may differ between patients that do and do not respond to checkpoint blockade.^19^

Despite the lack of data regarding specific bacterial species and mechanistic understanding of how the microbiome influences anti-tumor immunity, the improved ICI responses observed in preclinical models have garnered interest in clinical trials. Rationally manipulating the microbiome has proven complex. In a single-center study of patients treated for melanoma, those with higher fiber intake had better ICI responses, but those who used probiotics had lower alpha diversity, and were less likely to respond to ICI in a subset analysis.^128^ While broad over-the-counter probiotic administration in patients receiving ICI is to be avoided, trials of specific bacterial manipulation are ongoing, including using tailored probiotic administration (NCT03829111) and dietary modifications. Fecal transplant as a means to transfer the diverse bacterial ecosystem from responders to non-responders is also an area of great interest.^21^

## Direct modulation of tumor immunogenicity

In addition to indirect modulation of the tumor microenvironment, a number of modulators act more directly on the tumor to mediate immunogenicity, including chemotherapy, radiation, targeted therapy, and metabolic modifiers (Fig. 1).

### Chemotherapy

The primary function of cytotoxic agents is to reduce tumor burden by direct killing of tumor cells. Common chemotherapy agents can also have immunomodulatory properties that make them ideal partners to combine with immunotherapy. In destroying cancer cells, cytotoxic drugs release tumor-associated antigens that can stimulate a potent immune response creating an effect similar to vaccination. Additionally, chemotherapy drugs deplete immunosuppressive cells such as Tregs and MDSCs and enable expansion of tumor-specific Teff cells.^86,129^ Given this potential, considerable emphasis has been placed on exploring combinations of chemotherapy and ICI.

Mechanistic understanding of cytotoxic agents could facilitate rational combinations with immunotherapy approaches.^130^ For example, gemcitabine is a deoxycytidine analog that causes tumor cell apoptosis, resulting in antigen cross-presentation and cross-priming.^131^ Cyclophosphamide is an alkylating agent that has similar effects on antigen presentation and potently suppresses Tregs, while allowing homeostatic proliferation of antigen-specific Teff cells.^129,131,132^ Thus, cyclophosphamide is given prior to adoptive T cell therapy and oncolytic virotherapy to deplete non-specific lymphocytes and suppressive immune cells as well as to facilitate uptake and proliferation of the tumor antigen-specific effector cells.

In 2018, the first chemotherapy combination with ICI was approved by the FDA; carboplatin, pemetrexed, and pembrolizumab are now routinely used as a first-line therapy for non-small cell lung cancer. Similarly, the combination of nab-paclitaxel and atezolizumab (anti-PD-L1) was recently approved for triple-negative breast cancer based on a PFS benefit over chemotherapy in the Impassion130 trial.^133^ These successes have fueled interest, and chemotherapy plus immunotherapy trials are widely available for many cancer subtypes. In designing and interpreting such trials, one must consider the mechanism of specific chemotherapeutic agents, dose, and schedule to optimize combinatorial approaches.

### Radiation

Similar to chemotherapy, the primary goal of radiation is to directly kill tumor cells, and in so doing, tumor antigens are released, promoting an immune response. Radiation also enhances antigen presentation as well as TIL infiltration via inflammatory cytokines.^134^ The initial innate immune response reflects recognition of radiation-induced DNA damage within the cell, with macrophages subsequently eliciting migration of Teff cells to the tumor.^134,135^

Within the context of immunotherapy, radiation has garnered special attention with respect to the so-called “abscopal effect”, whereby radiation to one site of disease may induce a broader systemic anti-cancer response. In one such case, a patient with metastatic melanoma was treated with radiation therapy for a paraspinal mass while on ipilimumab and subsequently showed regression of multiple foci outside of the radiation field.^136^ Investigation into systemic effects demonstrated elevated antibody titers against NY-ESO-1 and increased activated CD4^+^ cells. These observations, along with recognition of improved survival following PD-1 blockade with pembrolizumab among patients with non-small cell lung cancer who had received prior radiation,^137^ has motivated significant interest in combining radiation with immunotherapy strategies.

To date, studies have been limited by small sizes, a variety of dosing regimens, and variable methods of sequencing immunotherapy and radiation. Nonetheless, these trials represent proof-of-concept.^135,138^ For example, in the PACIFIC trial, over 700 patients with non-small cell lung cancer were randomized to the PD-1 inhibitor durvalumab versus placebo after definitive chemoradiotherapy, and the patients who received ICI demonstrated improved clinical outcomes.^139^ Trials of ICI and radiation are enrolling a variety of cancer subtypes, and there are ongoing clinical trials of radiation with other immunotherapy approaches, such as a phase II study of radiation plus T-VEC (NCT02819843).

### Metabolism

An important aspect of any tumor’s interactions with its local microenvironment is the uptake and processing of nutrients and the excretion of cellular waste. Many of the nutrients that fuel tumor cells are also central to immune cells, complicating efforts to therapeutic target metabolic pathways. Nonetheless, certain aspects of the nutrient composition of the microenvironment may subtly favor tumor cell proliferation or immune cell activation, including in instances in which tumor cells are more dependent upon a specific nutrient than the immune cells that fight them.^140^ Key metabolic targets include glucose, amino acids, fatty acids, and lactate.

Termed the Warburg effect, scientists have long recognized the aberrant processing of glucose and lactate in tumor cells. Tumors are able to carry out rapid glycolysis under aerobic conditions, and in turn, they excrete lactate into the microenvironment. Depletion of glucose from the microenvironment and resultant acidification by lactate, coupled with the inability of dysfunctional tumor vasculature to remove H^+^ ions quickly, favors immune tolerance by causing TIL dysfunction, suppressing cytokine production, and promoting the accumulation of tolerogenic cells.^141,142^ These conditions are associated with G-CSF and GM-CSF excretion by local mesenchymal stem cells and upregulation of colony-stimulating factor production by the tumor that in turn leads to MDSC infiltration, with lactate additionally prompting upregulation of hypoxia-inducible factor promoting the M2-like macrophage phenotype.^140,143^ In cell line models, MDSC suppression can be achieved by inhibition of glycolysis and hence of G-CSF and GM-CSF release.^144^ Moreover, increased glucose consumption may favor a Treg phenotype, while TLR8 signaling blocks glucose metabolism favoring effector functions.^145^

A number of therapeutic strategies have shown preclinical efficacy in altering the interaction of the tumor with microenvironmental glucose, including by diminishing glucose availability, modifying glycolytic pathways, and/or changing lactate metabolism. The use of anti-diabetic drugs, particularly biguanides like metformin and phenformin that activate the energy-regulating AMPK pathway are one such approach.^146,147^ Phenformin was previously taken off the market due to a risk of lactic acidosis, but it has demonstrated anti-tumor efficacy in preclinical testing in both solid and hematologic malignancies.^148,149^ In melanoma models, phenformin decreases MDSCs and increases ICI efficacy.^150^ These drugs may have a variety of effects beyond their impact on glucose metabolism, including altering local angiogenesis^147^ and inhibiting the MAPK pathway through AMPK activation.^148^ This impact on the MAPK pathway has made RAF-driven tumors a particularly attractive target, and phenformin is currently being studied in the phase I setting together with the BRAF and MEK inhibitors dabrafenib and trametinib in patients with BRAF V600E mutant melanomas (NCT03026517).

mTOR inhibitors have also been used to modulate glycolysis,^140^ although their use to mediate tumor interaction with microenvironmental glucose is complicated by the fact that these inhibitors have pleiotropic effects on numerous immune cells and vasculature.^151^ mTOR’s role in regulating CD8^+^ T cell differentiation and function, as well as the impact of inhibition on these cells, is an active area of research. While some studies suggest that mTOR inhibition may promote memory function, other data suggest that T cells lacking mTOR differentiate into tolerogenic Tregs.^152,153^ Given the complex impact of mTOR inhibition in vivo, ex-vivo inhibition of the AKT-mTOR pathway during adoptive T cell therapy may hold promise.^154^

Reductions in lactate, via modulation in production or enhancements in clearance, are being investigated. Selective targeting of monocarboxylate transporters (MCTs) has been proposed, particularly for highly glycolytic tumor models, to halt lactate excretion into the immune microenvironment.^155^ MCT1 inhibition, using AZD3965, is currently under phase I investigation (NCT01791595). LDHA inhibitors, which mediate lactate production, are an exciting new class of agents that have demonstrated preclinical efficacy,^156^ and we expect to see clinical development of these drugs.

Amino acids are another key target, including the recently popular approach of modulating tryptophan availability. Treg activity and MDSC recruitment correlates with tryptophan breakdown, in part mediated by indoleamine 2,3-dioxygenase (IDO).^157^ A variety of IDO inhibitors are currently in phase I-III clinical trials together with checkpoint inhibitor therapies. In a widely publicized study, the ECHO-301/KEYNOTE-252 trial of the IDO inhibitor epacadostat plus pembrolizumab for advanced melanoma failed to meet its primary endpoint of altering PFS. However, biomarkers for response are unknown,^158^ and it may still be that certain patients with high microenvironmental tryptophan and/or more robust IDO inhibition will benefit from IDO-targeted therapy.^159^

Arginine metabolism has also been the focus of significant therapeutic interest. Certain tumors such as liver cancers and melanoma cannot synthesize arginine and may rely on environmental sources. ADI-PEG 20 monotherapy, which depletes arginine, has shown safety but limited efficacy to date.^160,161^ While initial studies of arginine focused on the necessity of this amino acid for tumor cells, an increasingly robust understanding of its importance to immune cells has motivated novel study designs.^162^ Investigations of ADI-PEG-20 in combination with ICI are underway.

In contrast to studies aimed at depleting arginine, attempts to increase arginine levels to promote Teff response are ongoing. One of the means by which tolerogenic cells inhibit T cell responses is by degradation of arginine that would otherwise be used by Teff cells.^163^ Inhibition of arginine degradation is therefore also being studied in combination with ICI in an early phase trial for patients with melanoma (NCT02903914).

An additional area of interest has been the role of adenosine in promoting an immunosuppressive microenvironment. Apoptosis of Tregs leads to the release of ATP, which is used by CD39 and CD73 to make adenosine.^164^ Adenosine, in turn, appears to have an immune suppressive effect, raising concerns that Treg apoptosis can paradoxically potentiate immune tolerance.^164^ Therapeutic strategies directed at suppressing adenosine have included targeting CD39 and CD73 or blockade of the A2A adenosine receptor, with research ongoing.^165^

The amino acid cysteine may also play an important role in mediating CD8^+^ T cell-induced ferroptosis, the mechanism by which abnormal lipid metabolism within a tumor cell induces cell death. Tumor cell uptake of cystine is impaired by interferon-gamma produced by CD8^+^ T cells.^166^ Cystine levels, in turn, appear to mediate glutathione activity, which helps to inhibit lipid oxidization and ferroptosis.^167^ When cyst(e)inase is used to degrade cystine, tumor cells are more likely to undergo immune-mediated ferroptosis. Moreover, decreased levels of SLC3A2, which forms part of the cellular glutamate-cystine antiporter, appear to correlate with improved responses to anti-PD-1 therapy, offering the rationale for the development of combination immunotherapy together with treatments aimed at altering cysteine metabolism.^166^ Both immunotherapy- and radiotherapy-mediated immune responses may also partially derive from regulation of SLC7A11, an additional component of the antiporter.^166,167^

Lipids are yet another metabolic target in the microenvironment. Lipid-laden MDSCs tend to be more tolerogeneic.^168^ Furthermore, cholesterol has been hypothesized to mediate interactions between immune effector cells and tumors, and inhibition of cholesterol esterification increased CD8^+^ T cell proliferation in preclinical studies. This therapeutic strategy has also shown in vivo anti-melanoma effects using the anti-atherosclerotic drug avasimibe.^169^ Additional preclinical studies indicate that pharmacologic increases in fatty acid oxidation are associated with enhanced benefit from ICI, creating potential novel avenues for future combination therapies.^170^ In another approach, the endoplasmic reticulum XBP1 oxidative stress factor alters dendritic cell functioning necessary for T cell-mediated immune responses by modulating lipid metabolism.^171^

### Targeted therapy

Similar to chemotherapy, targeted therapies promote cytoreduction with concomitant modification of tumor antigenicity. Tumors are exquisitely sensitive to appropriate targeted therapies, but response is almost always followed by resistance. However, by understanding how targeted therapies modify the tumor microenvironment, and optimizing combinations with immunotherapy, there is potential to convert these potent transient responses to durable benefit.

Many targeted therapies alter the balance of local immune cells and several also impact vasculature. One of the best-studied examples of the dynamic effects of targeted therapy on the microenvironment is with the use of BRAF and MEK inhibitors for melanoma. Combined BRAF and MEK inhibition in patients with V600 mutations in the *BRAF* gene exhibits response rates as high as 87%.^172^ Beyond their direct anti-tumor effects, BRAF plus MEK inhibition upregulates expression of MHC and melanoma differentiation antigens, including gp-100 and MART-1.^173^ In turn, exposed tumors have higher infiltration of antigen-specific T cells, APCs, and inflammatory cytokines, in conjunction with decreased vascular endothelial growth factor (VEGF).^174^ BRAF inhibitors specifically have been associated with decreased infiltration of tolerogenic immune cells, such as MDSCs and Tregs.

These favorable effects are dynamic. Within two weeks of exposure to BRAF/MEK inhibitor therapy, in vitro studies suggest that tumor cells paradoxically downregulate melanoma differentiation antigens, with apparent decreases in T cell recognition.^175^ Biopsies from patients treated with BRAF inhibitors show that both PD-1 and TIM-3, markers of immune exhaustion, are upregulated at the time of tumor progression.^176^ Given these time-dependent changes in the immune microenvironment, sequencing of drug combinations may be critical.

At present, rational strategies for using targeted therapies to augment immune response represents one of the most active areas of clinical research. A recent phase II randomized trial of patients with BRAF V600^E/K^ mutant advanced melanoma demonstrated improved PFS (though did not reach its pre-specified endpoint) and duration of response in patients treated with dabrafenib plus trametinib and pembrolizumab versus those treated with dabrafenib plus trametinib and placebo.^177^ The COMBI-I trial, investigating dabrafenib, trametinib, and the anti-PD-1 agent PDR001 in patients with advanced BRAF V600 mutant melanoma has yielded promising preliminary results, reporting a 94% disease control rate and a 33% complete response rate;^178^ the full results of these trials are eagerly awaited.

Many targeted therapies also modulate tumor PD-L1 expression, further motivating combination therapies. For example, PARP inhibitors have been associated with increased PD-L1 expression,^179^ giving impetus to the JAVELIN BRCA/ATM study of PARP inhibition together with the PD-L1 inhibitor avelumab.^180^ Anti-HER2 therapy also has been associated with upregulation of PD-L1 expression, enhanced antigen presentation, and indirect activation of both the innate and adaptive immune systems,^181^ leading to studies of combined anti-HER2 treatment plus ICI across a number of disease sites.^182,183^

Despite the theoretical benefits of such combinations for promoting anti-tumor efficacy, combinations of immunotherapy with targeted agents come with significant risk of toxicity. In melanoma, combinations of dabrafenib, trametinib, and anti-PD-1 have led to higher rates of grade 3/4 adverse events than would be expected for targeted therapy alone.^177,178^ Hepatotoxicity, in particular, has emerged as an important consideration across numerous studies combining immunotherapy with molecularly targeted therapy, either concomitantly or sequentially.^173,184,185^

Targeted therapies may also play a role in altering the tumor endothelium, allowing T cell and NK cell infiltration, and tolerogenic cell infiltration may be decreased.^186–189^ Combination trials of VEGF-targeting therapy plus ICI have been fruitful. The VEGF receptor tyrosine kinase inhibitor axitinib plus anti-PD-(L)1 recently demonstrated improved OS and PFS for patients with advanced renal cell carcinoma compared to sunitinib, leading to FDA approval of two such combinations.^190,191^ Similarly, lenvatinib plus pembrolizumab was granted accelerated approval for patients with advanced endometrial cancers.^192^ These studies emphasize the importance of the tumor vasculature in mediating immune cell infiltration, and we expect that pharmacologic and non-pharmacologic mediators of tumor vasculature will continue to garner interest in combination with both immune checkpoint inhibition and adoptive T cell therapy.^193^

## Conclusion

The last decade has seen a shift in the care of cancer patients from a focus on cytotoxic therapies toward approaches that enhance anti-tumor immunity. Immunotherapy has extended the lives of cancer patients worldwide, but most patients still do not achieve durable disease control. In this review, we have described our vision for the next frontier of this field involving strategies aimed at both direct modification of tumors and indirect modification of the microenvironment to sensitize resistant tumors to immunotherapy. We acknowledge that there is overlap and interplay between direct and indirect modification strategies, but we believe that this distinction in terms of the primary goal of a therapy is useful for understanding rational combinatorial strategies. Better defining contributors to an immunogenic microenvironment constitutes an important first step, including thorough investigations not only of local immune cell infiltration but also of such modifiable factors as the gut microbiome, body mass index, and vascular supply to the tumor. Current immunotherapy approaches are simply the tip of the proverbial iceberg. What lies beneath is a complex environment that supports the tumor, and we expect that targeting this foundation will yield the next breakthroughs in cancer immunotherapy.
